# Etazene induces developmental toxicity in vivo* Danio rerio* and in silico studies of new synthetic opioid derivative

**DOI:** 10.1038/s41598-021-03804-9

**Published:** 2021-12-20

**Authors:** Łukasz Kurach, Agnieszka Chłopaś-Konowałek, Barbara Budzyńska, Marcin Zawadzki, Paweł Szpot, Anna Boguszewska-Czubara

**Affiliations:** 1 Independent Laboratory of Behavioral Studies, Medical University of Lublin, 4A Chodzki Str., 20-093 Lublin, Poland; 2 Institute of Toxicology Research, 45 Kasztanowa Str., 55-093 Borowa, Poland; 3 Department of Forensic Medicine, Wroclaw Medical University, 4 J.Mikulicza-Radeckiego Str., 50-345 Wrocław, Poland; 4 Department of Medical Chemistry, Medical University of Lublin, 4A Chodzki Str., 20-093 Lublin, Poland

**Keywords:** Toxicology, Risk factors

## Abstract

Synthetic opioids are gaining more and more popularity among recreational users as well as regular abusers. One of such novel psychoactive substance, is etazene, which is the most popular opioid drug in the darknet market nowadays. Due to limited information available concerning its activity, we aimed to characterize its developmental toxicity, including cardiotoxicity with the use of in vivo* Danio rerio* and in silico tools. Moreover, we aimed, for the first time, to characterize the metabolite of etazene, which could become a potential marker of its use for future forensic analysis. The results of our study proved severe dose-dependent developmental toxicity of etazene (applied concentrations 10–300 µM), including an increase in mortality, developmental malformations, and serious cardiotoxic effects, as compared with well-known and used opioid—morphine (applied concentrations 1–50 mM). In silico findings indicate the high toxic potential of etazene which may lead to drug-drug interactions and accumulation of substances. Furthermore, phase I metabolite of etazene resulting from N-dealkylation reaction was identified, and therefore it should be considered as a target for toxicological screening. Nonetheless, the exact mechanism of observed effects in response to etazene should be further examined.

## Introduction

**Figure 1:**
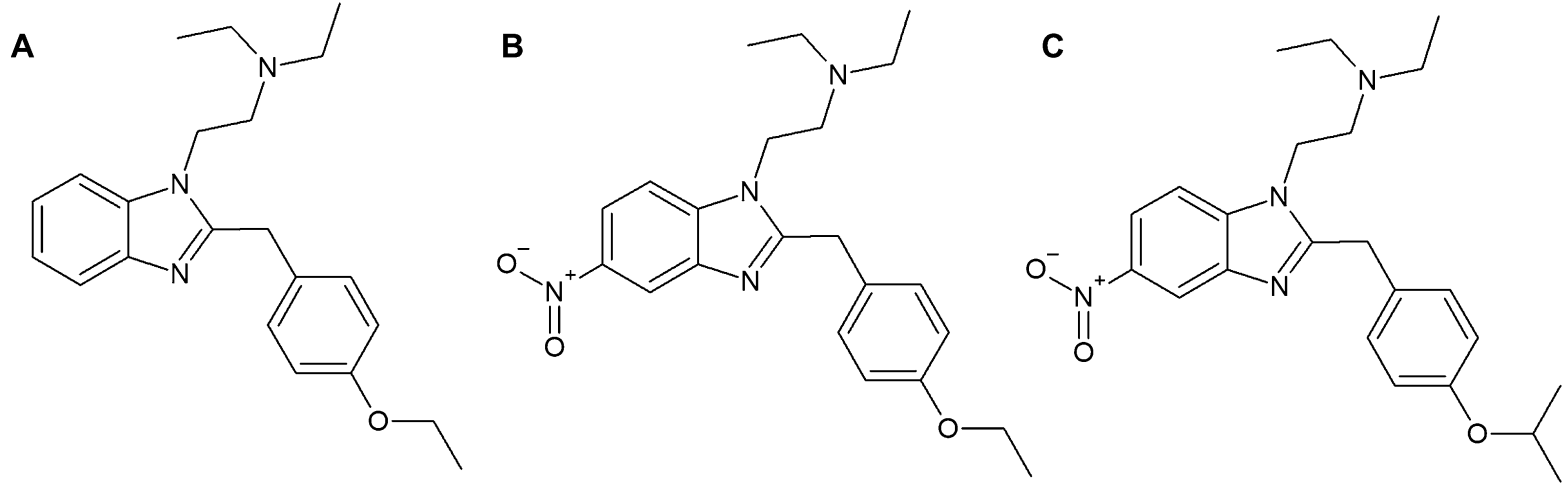
Chemical formula of (**A**) etazene, (**B**) etonitazene, and (**C**) isotonitazene.

Novel psychoactive substances (NPS) have risen in popularity, especially among teenagers and young adults. One of the rapidly growing classes of NPS is the group of synthetic opioids (SO). According to the annual report of the European Monitoring Centre for Drugs and Addiction (EMCDDA), 59 of SO appeared on the European market since 2009, while at the end of 2019, only 2 out of 9 reported new opioids, were fentanyl derivatives^1^. One of them was etazene (ETZ) [2-(2-(4-ethoxybenzyl)-1H-benzo[d]imidazol-1-yl)-N,N-diethylethan-1-amine], which appears to be the most popular opioid drug in the darknet market^2^. The first synthesis of ETZ and its homologs (Fig. 1) dates back to the 1950s, intending to develop better and safer analgesics^3^. Pharmacological evaluation has shown that they are several orders of magnitude stronger than morphine (MORPH) in their antinociceptive action. ETZ appeared to be one of the strongest (70 × stronger than MORPH) benzimidazole derivatives^4^. Although ETZ recently gained popularity as a recreational drug on the illegal market, there is limited information concerning its activity.

The *Danio rerio* embryo is a new toxicological model with increasing popularity due to its high throughput, low cost, which serve as complementary to rodents. The rapid development of zebrafish (ZF) larvae allows for the assessment of both, the acute and chronic effects of substances on their organism. The transparency of the larvae allows tracking drug-induced changes without the use of invasive techniques and enables the assessment of neurotoxicity, cardiotoxicity, hepatotoxicity, and potential developmental malformations^5^. It is noteworthy to mention that 70% of the human genome, physiology, and structure of organs are similar to ZF^6^. The *D. rerio* model has been frequently used in studies of NPS, including behavioral assessment of opioid addiction, anxiety, memory, and toxicological and pharmacological targets^7^. Recently, the *D. rerio* model has been successfully used to identify metabolites for various NPS groups, including cannabinoids^8^, opioids^9^, and cathinones^10^. Comparing metabolic processes of *D. rerio* to humans, consistent results have been obtained, which may be the basis for replacing the existing in vitro methods, characterized by substantial limitations^11^.

The opioid system has been well characterized in *D. rerio*. It has been revealed that the *D. rerio* opioid receptors share a high degree of similarity with those from mammals. In situ hybridization studies at 24 hpf (hours post fertilization) of *D. rerio* larvae show that the opioid receptors are widely distributed in its central nervous system (CNS) and they are analogs of three human subtypes receptors, δ (zfDOR1, zfDOR2), µ (zfMOR), κ (zfKOR)^12^.

Therefore, the aim of our research was to characterize developmental toxicity according to Fish Embryo Acute Toxicity (FET) Test protocol, including acute cardiotoxicity of the new synthetic opioid—ETZ, using the *D. rerio* larvae model in vivo, and in silico tools. The MORPH was used as a reference drug in FET. Moreover, we aimed to characterize, for the first time, the metabolite of ETZ, which could become a potential marker of use for future forensic analysis.

## Results

### In vivo developmental toxicity of ETZ and MORPH in FET

**Figure 2:**
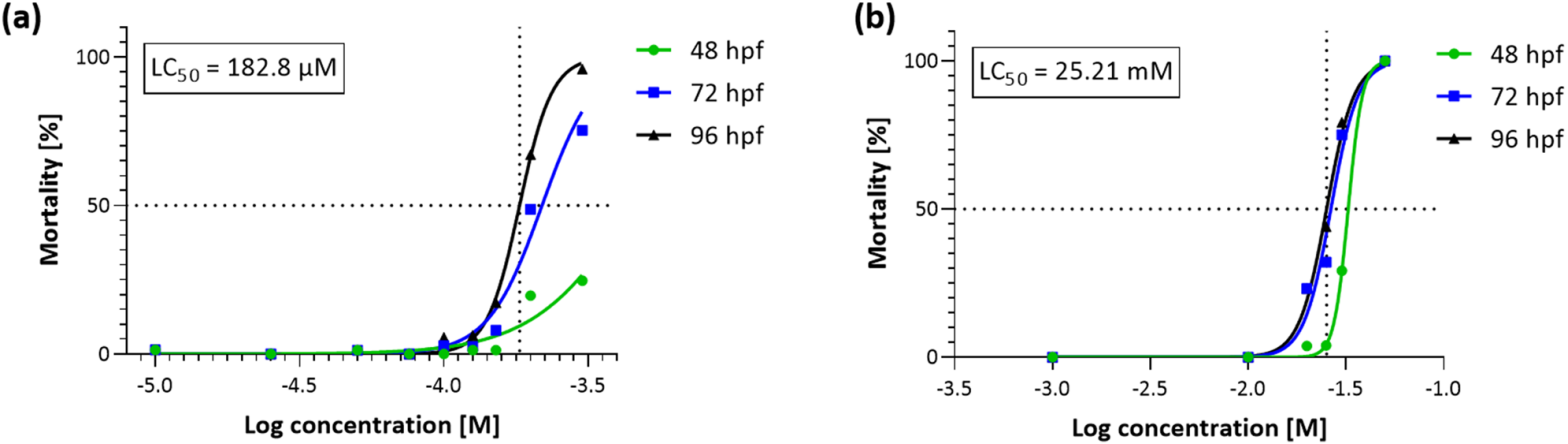
Mortality of *D. rerio* larvae in a time- and a concentration-dependent manner to ETZ (**a**) and MORPH (**b**) exposure. The median lethal dose (LC_50_) was determined based on cumulative mortality obtained from three independent experiments (n = 24) at 96 hpf using nonlinear, four parameters regression analysis.

Figure 2 shows the effect of ETZ and MORPH in the FET test. Exposure to both compounds induced concentration- and time-dependent mortality effect. The LC_50_ at 96 hpf was determined to be 182.8 μM (95% confidence interval: 178.5–187.4 μM, R = 0.9966) and 25.21 mM (95% confidence interval: 23.89–26.53, R = 0.9927) for ETZ and MORPH, respectively. LC_50_ of ETZ is approximately 135 × fold higher than MORPH in the *D. rerio* FET test. Survival at negative control was higher than 95% at the end of the test.

Table 1 represents the *D. rerio* hatching rate during ETZ exposure at 72 hpf (one-way ANOVA F(3,11) = 95.86, *p* < 0.0001) and 96 hpf (one-way ANOVA F(3,11) = 81.94, *p* < 0.0001). Post hoc Dunnett test showed that, following exposure to 25 μM of ETZ, the hatching rate decreased to 36% (*p* < 0.0001) and reached 0% (p < 0.0001) in 50 μM treated *D. rerio* at 72 hpf. At 96 hpf, hatching rate decreased from 47 (*p* < 0.001) to 3% (*p* < 0.0001) with increasing ETZ exposure concentration. In the negative control group, 100% of *D. rerio* embryos were hatched within 72 hpf.

**Table 1:** Hatching rate (%) during ETZ exposure at 72 and 96 hpf obtained from three independent experiments.

Concentration [µM]	0	10	25	50
**ETZ**				
72 hpf	97.3 ± 1.9	97.3 ± 1.9	36 ± 13.6****	0****
96 hpf	100	100	47.3 ± 14***	3 ± 4.2****

Data are shown as means ± SD; ***p < 0.001, ****p < 0.0001 versus negative control group (n = 24); Dunnett’s test.

As shown in Table 2, MORPH has no effect on the hatching process during the FET experiment. One-way ANOVA did not show any effect neither 72 hpf (F(4,14 = 0.7806, *p* = 0.5627) nor 96 hpf (F(4,14) = 1, *p* = 0.4516).

**Table 2:** Hatching rate (%) during MORPH exposure at 72 and 96 hpf obtained from three independent experiments (n = 24 ).

Concentration [µM]	0	1	10	20	25
**MORPH**					
72 hpf	100	96 ± 5.7	98.7 ± 1.9	98.3 ± 2.4	83.3 ± 23.6
96 hpf	100	100	100	100	97.6 ± 3.3

Data are shown as means ± SD.

### ETZ- and MORPH-induced changes in heart rate and cardiotoxicity in *D. rerio* embryos

**Figure 3:**
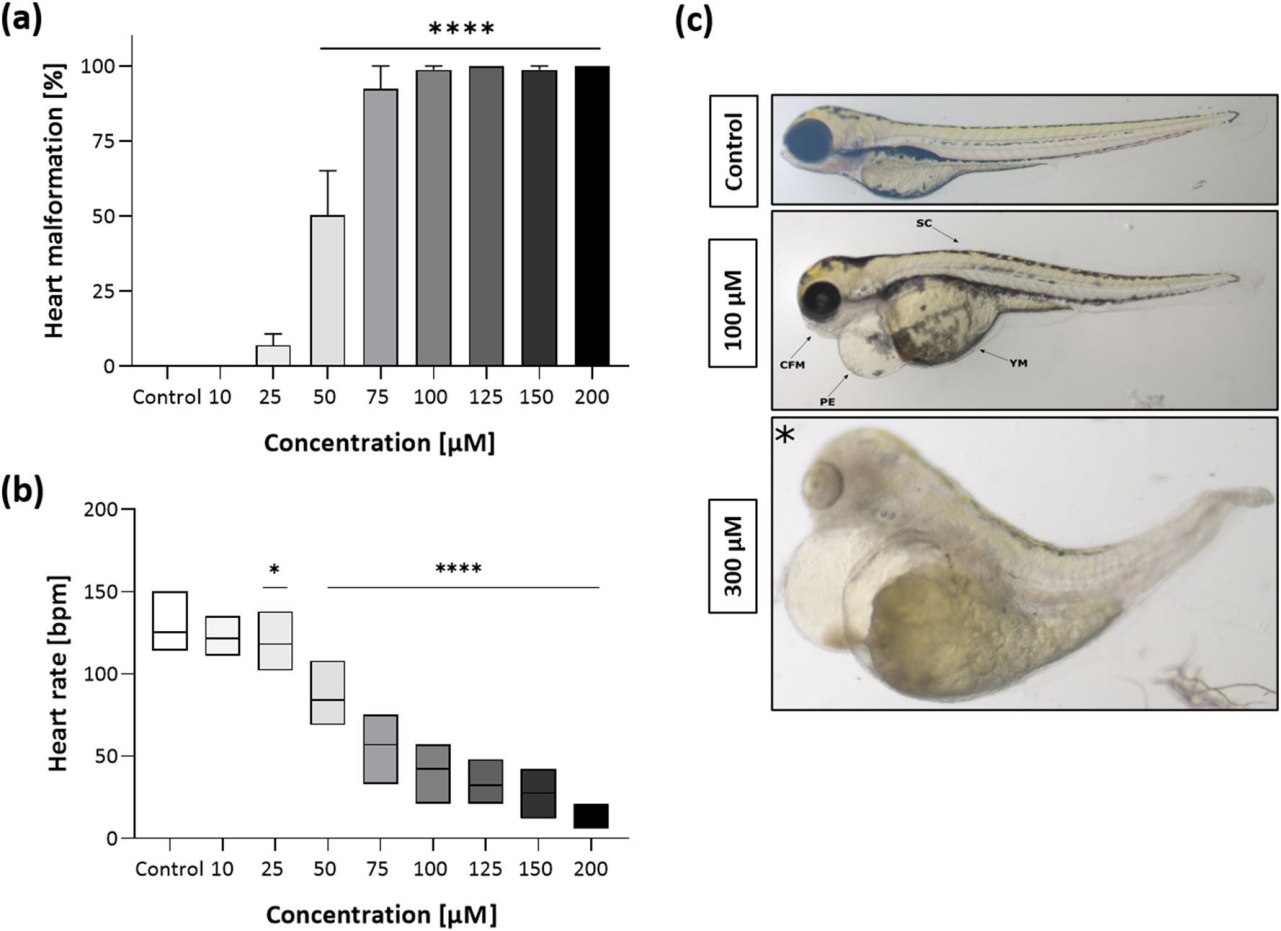
Sublethal endpoints after 96 hpf ETZ exposure; (**a**) heart malformation at 96 hpf; (**b**) heart rate estimated by beats per minute of a 96 hpf larvae from three independent experiments; (**c**) representative morphological alterations in 96 hpf larva at 100 and 300 µM are indicated by arrows, CFM—craniofacial malformation, PE—pericardial edema, SC—spinal curvature, YM—yolk sac malformation; *general developmental malformations. Data are presented as means ± SD; one-way ANOVA, n = 10; *p < 0.05, ****p < 0.0001 versus negative control group; Dunnett’s test.

Figure 3a presents the sublethal effects of ETZ treatment at 96 hpf (one-way ANOVA F(8,26) = 65.7, *p* < 0.0001). Post hoc Dunnett test revealed significant heart malformation after 50–200 μM (*p* < 0.0001) ETZ exposure. The normal heart rate of 96 hpf *D. rerio* was significantly reduced (one-way ANOVA F(8,239) = 579, *p* < 0.0001) by 7.5% upon 25 μM (*p* < 0.05) (Fig. 3b) and down to 13.3% of negative control value after 200 μM treatment. Furthermore, we observed craniofacial malformation, yolk edema, deformed body shape at the concentration of ETZ higher than 100 μM (Fig. 3c). At the concentration of 200 μM we observed a decrease in heart rate and general body malformation, lack of pigmentation, cylindrical heart with no visible blood flow. Due to the high mortality rate at the dose of 300 µM, the heartbeat was not assessed.

**Figure 4:**
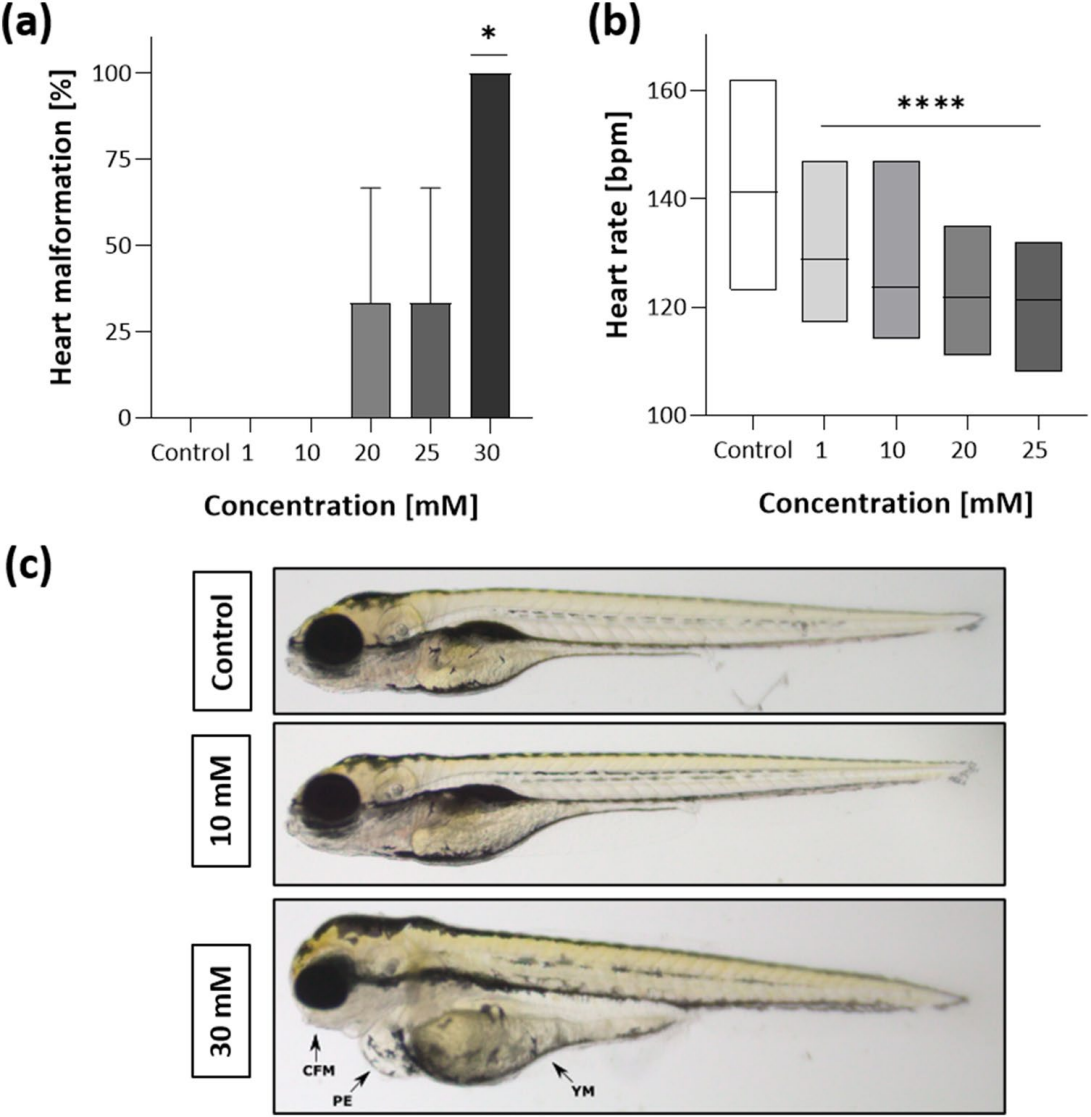
Sublethal endpoints after 96 hpf MORPH exposure; (**a**) heart malformation at 96 hpf; (**b**) heart rate estimated by beats per minute of a 96 hpf larvae from three independent experiments; (**c**) representative morphological alterations in 96 hpf larva at 10 and 30 mM are indicated by arrows, CFM-craniofacial malformation, PE-pericardial edema, YM-yolk sac malformation; Data are presented as means ± SD; one-way ANOVA, n = 10; *p < 0.05, **** *p* < 0.0001 versus negative control group; Dunnett’s test.

Heart alteration caused by MORPH are presented at Fig. 4a (one-way ANOVA F(5,17) = 4.1, *p* = 0.021), Dunnett’s test revealed significant heart malformation after 30 mM exposure (*p* = 0.0127). Heartbeat (Fig. 4b) was significantly affected by MORPH (one-way ANOVA F(4,149) = 38.66, *p* < 0.0001) at the concentration of 1 mM and higher (*p* < 0.0001). Representavive malformations are presented at Fig. 4c.

**Figure 5:**
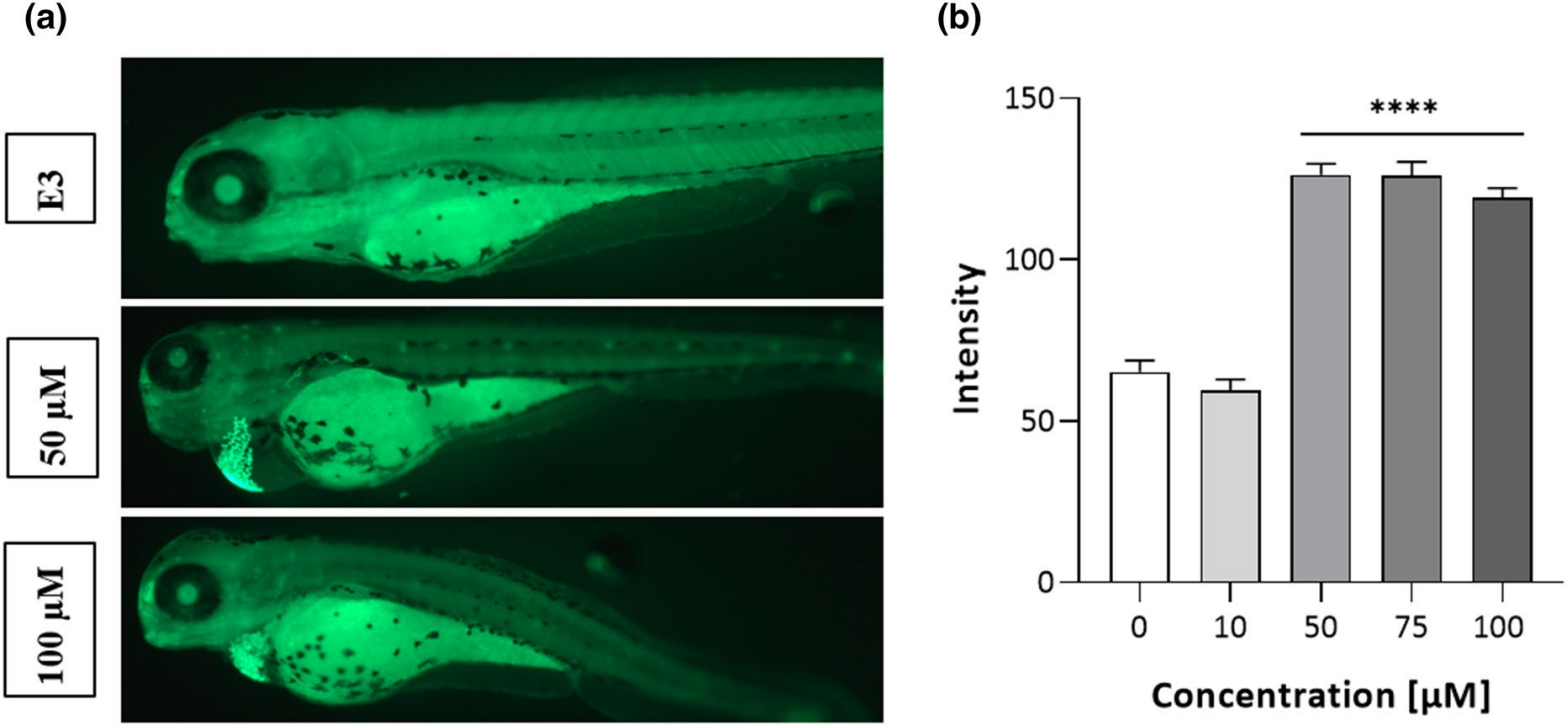
Apoptosis evaluation in 96 hpf ETZ-exposed zebrafish larvae, (**a**) representative images of 96 hpf larvae at 50 and 100 µM; (**b**) quantitative analysis of the relative intensity of apoptotic cells. Data are presented as means ± SD; one-way ANOVA, n = 3 in three independent experiments; ****p < 0.0001; Dunnett’s test.

AO staining (Fig. 5) revealed a cluster of apoptotic cells localized below the heart, within edema boundaries. The quantitative analysis (Fig. 5) showed a significant increase of relative fluorescence intensity at 50–100 µM (one-way ANOVA F(4,44) = 88.24; Dunnett's test, *p* < 0.0001).

### Acute exposure of ETZ induces arrhythmia

**Figure 6:**
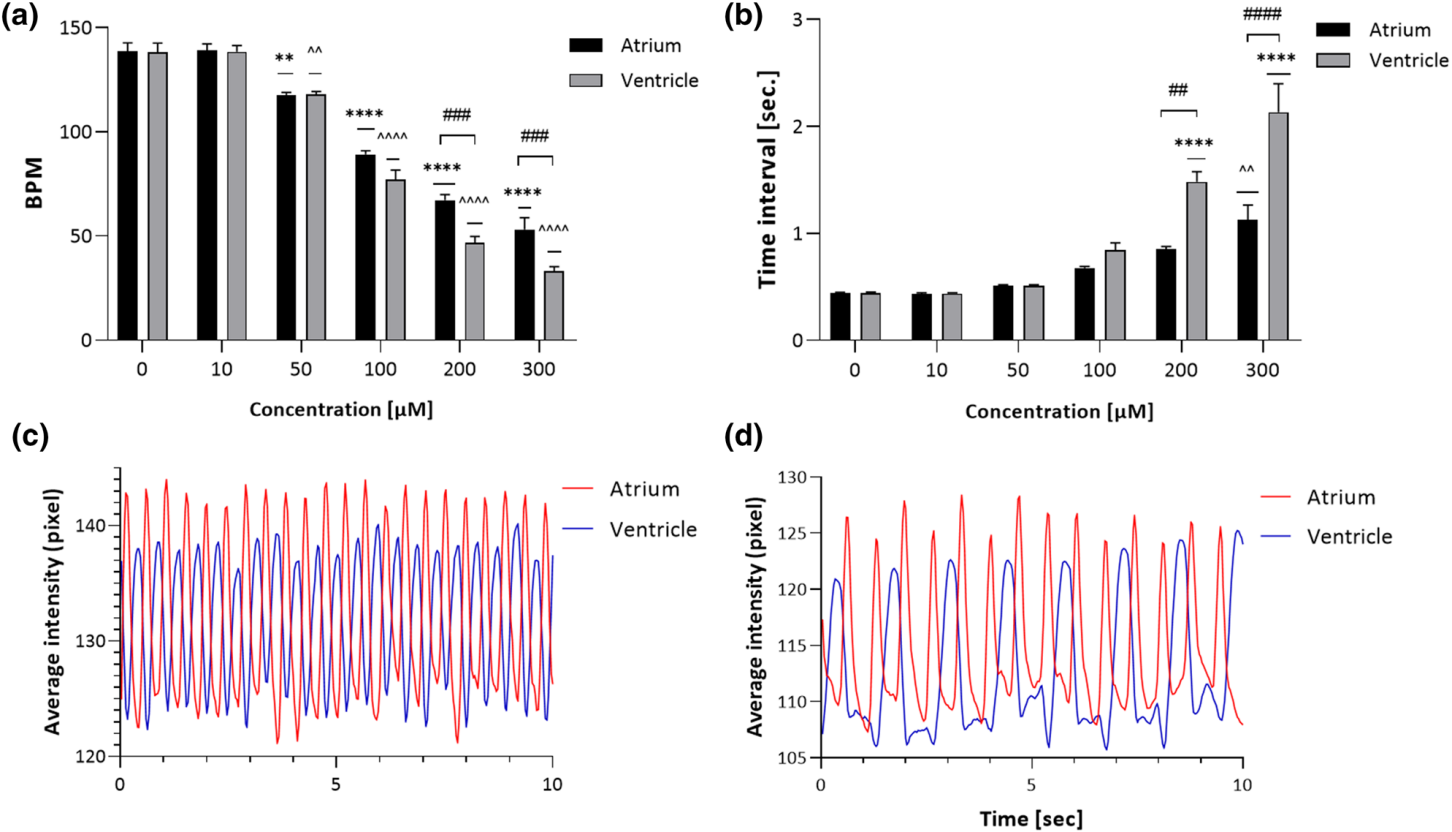
Effect on (**a**) BPM and (**b**) heart rhythmicity after 40 min. incubation with different concentrations of etazene (ETZ). The dynamic pixel change pattern in the atrium and ventricle in control (**c**) and ETZ (**d**). Data are presented as means ± SEM, two-way ANOVA, n = 5–7 in three independent experiments; **p < 0.01, ****p < 0.0001 versus negative control; ^^*p* < 0.01, ^^^^*p* < 0.0001 versus negative control; ##*p* < 0.01, ###*p* < 0.001, ####*p* < 0.00001 atrium versus ventricle; Tukeys test.

Initial studies of acute time- and concentration-dependent ETZ administration revealed cardiac arrhythmias induced by concentrations of 200 and 300 µM (Fig. S1). The main study revealed that after 40 min of incubation of ETZ at various concentrations, a dose-dependent decrease in the number of atrial and ventricular contractions was observed (two-way ANOVA: concentration [F(5,202) = 292.5, *p* < 0.0001], contraction atrium/ventricle [F(1,202) = 19.82, *p* < 0.0001], and interactions effect [F(5,202) = 4.337, *p* = 0.0009) (Fig. 6a). The post hoc Tukey’s test revealed that ETZ 50 µM significantly decreased contraction of atrium (*p* = 0.0033), ventricle (*p* = 0.0048); ETZ 100–300 µM significantly decreased contraction of atrium and ventricle (*p* < 0.0001) as compared with negative control group, while number of contractions of ventricle relative to atrium were significantly lower after exposure of ETZ 200 µM (*p* = 0.0005) and ETZ 300 µM (*p* = 0.0007). The beat-to-beat interval analysis of the atrium and ventricle were in a 1:1 ratio up to a concentration of 100 µM (Fig. 6c). Higher concentrations induced arrhythmia (two-way ANOVA: concentration [F(5,202) = 292.5, p < 0.0001], beat-to-beat interval [F(1,202) = 19.87, *p* < 0.0001], and interactions effect [F(5,202) = 7.969, *p* < 0.0001]) (Fig. 6b), characterized by a prolongation of contractions time of ventricle in relation to atrium caused by ETZ 200 µM (*p* = 0.0011) and ETZ 300 µM (*p* < 0.0001) according to Tukey’s analysis. The same post hoc test indicated that time interval of atrium was significantly decreased compared to negative control after ETZ 300 µM treatment (*p* = 0.011), whereas time interval of ventricle was lower compared to negative control after ETZ 200 and 300 µM treatment (*p* < 0.0001). The majority were disorders characterized by prolongation of ventricular systole phase relative to the atrium (Fig. 6d). Sample videos of the described rhythm disturbances shown in Fig. 5c, d are provided in supplementary materials (Video S1,S2).

### ADME profiling

All predicted properties absorption, distribution, metabolism, excretion (ADME) are presented in Table 3. Lipophilicity is represented by the logP, which helps predict in vivo permeability through biological membranes. ETZ has been shown to have almost 3 times greater lipophilicity value than MORPH. The further analysis predicts that both compounds show high gastrointestinal absorption and the possibility to penetrate BBB. Only MORPH is P-gp substrate. Metabolite profiling revealed that MORPH is an inhibitor against CYP2D6, and ETZ for CYP: 1A2, 2C19, 2D6, 3A4. Druglikeness analysis did not show any violation and meet the range criteria described in used models, both compounds have a bioavailability score of 0.55 and are likely *per os* active in humans.

**Table 3:** Results of in silico ADME profiling of ETZ and MORPH.

	Physicochemical properties	Pharmacokinetics			CYP P450 inhibitor				
	cLogP	GIA	BBB	P-gp	1A2	2C19	2C9	2D6	3A4
ETZ	4.10	High	+	−	+	+	−	+	+
MORPH	1.41	High	+	+	−	−	−	+	−

cLogP: logarithm of compound partition coefficient between n-octanol and water, GIA: gastrointestinal absorption, P-gp: P-glycoprotein substrate. ( +) positive, ( −) negative.

### Metabolite identification

**Figure 7:**
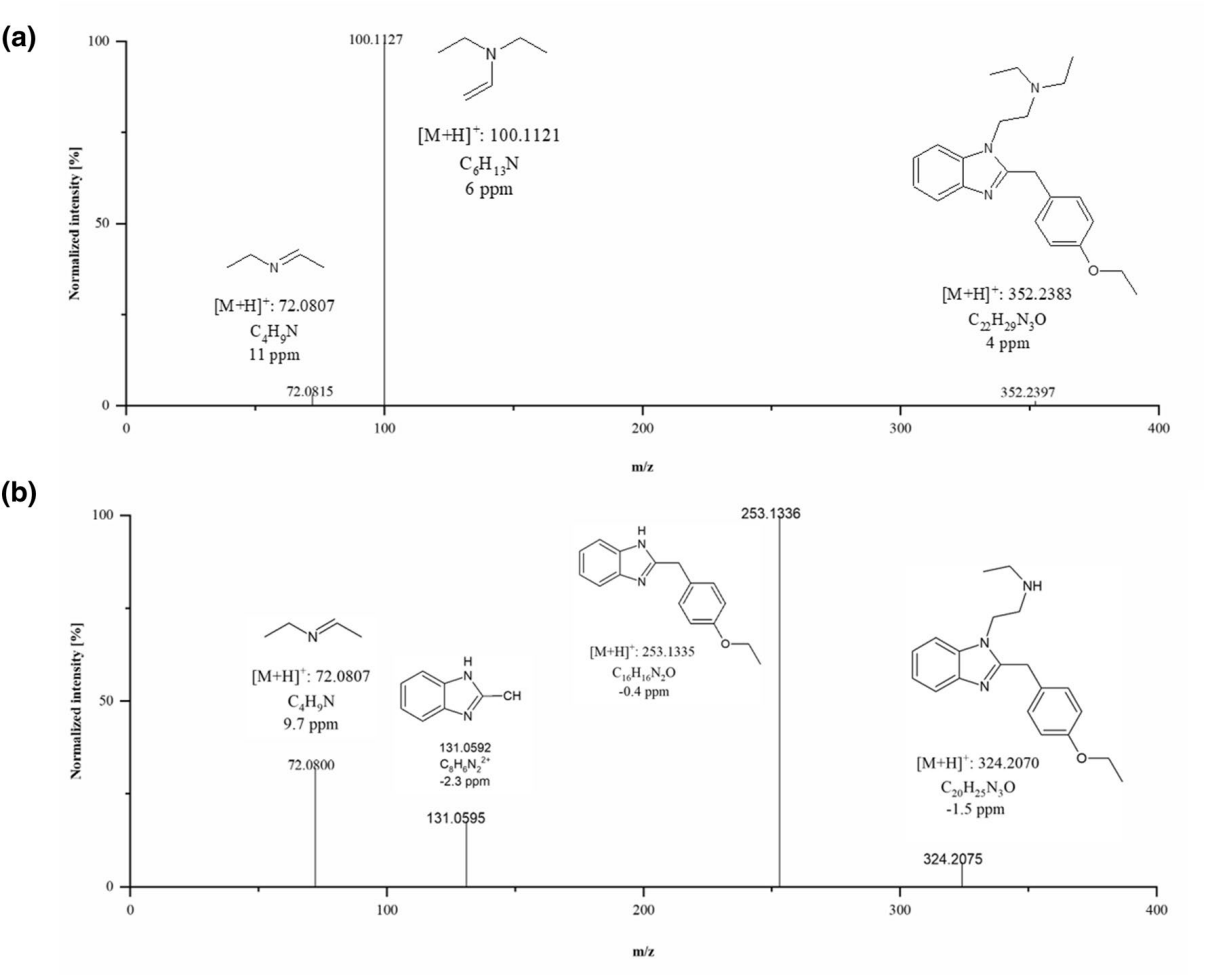
MS/MS spectra of (**a**) ETZ and (**b**) its metabolite identified in *D. rerio* model recorded using collision energy 20 eV.

ChemSketch 2018 2.5 (ACD/Labs, Toronto, Canada) was used to draw structures of hypothetical metabolites and to calculate their exact masses. Mass spectral interpretations were based on general fragmentation rules and metabolites were identified based on their precursor mass (PM), the calculated molecular formulae, and the fragmentation patterns compared to those of the parent compound and already known metabolites of ETZ homologs. Figure 7a represents MS/MS spectra of ETZ and Fig. 7b N-desethyl-etazene along with calculated exact masses, elemental composition, and mass deviation errors. Fragmentation patterns of both compounds have one similar ion at m/z 72, which corresponds to the imine fragment. The most abundant ion at m/z 100 corresponds to the diethylamine fragment resulting from the fragmentation of ETZ. N-dealkylation process of ETZ leads to the creation of its metabolite, which MS/MS spectrum revealed the most abundant ion at m/z 253, originated from the N-ethylethanamine fragment loss. Further fragmentation leads to a benzimidazole fragment creation. Fragmentation of ETZ metabolite at higher collision energy (40 eV) revealed additional fragments m/z 107, 195, 224. MS/MS spectrum of ETZ at higher collision energy does not possess any additional ions.

### Discussion

In the era of rapid development of psychoactive substances, it is crucial to understand the risks posed by NPS. The present study has been designed to evaluate for the first time toxicity of a newly introduced non-fentanyl synthetic opioid derivative—ETZ. A previous study revealed that, ETZ was assessed to be 70 times more potent than morphine in an animal model of analgesia^4^. The toxicological data for ETZ obtained by clinicians are not available, but β-arrestin/mini-Gi recruitment assay revealed high potency of ETZ (MOR-βarr2, EC_50_ = 54.9 nM; MOR-mini-Gi, EC_50_ = 164 nM) against MORPH (MOR-βarr2, EC_50_ = 338 nM; MOR-mini-Gi, EC_50_ = 385 nM)^13^. Current knowledge regarding its activity is available on the internet forums, where users describe the feeling of euphoria. Concerning prescribed opioids intake in humans, analgesic, euphoric effects, reduction of the body temperature, slow heart rate, a decrease of blood pressure, as well as respiration depression was observed^14^. Thus, it was necessary to evaluate the toxicological and pharmacological profile of ETZ compared to the known opioid MORPH.

Our study revealed strong developmental toxicity of ETZ at the micromolar concentration (LC_50 =_ 182.8 µM) in *D. rerio* embryos, in particular, morphological abnormalities, cardiotoxicity and delayed hatching. ETZ treatment at a concentration of 10 µM did not produce noticeable signs of sublethal endpoints during the FET test, whereas the concentration of 25 µM highly reduced the hatching rate at 72 hpf larvae. Hatching is a critical period of *D. rerio* embryo development and occurs at approximately 72 hpf. The hatching process is described as the combined effect of the activity of hatching enzyme released by the embryo’s hatching gland cells to weaken the chorion and then the mechanical tearing of egg envelopes. It occurs when the embryo reaches a size, at which its energy requirements for oxygen exceed the diffusion capabilities of this gas through the egg envelopes and perivitelline fluid^15^. Factors such as the inhibition of hatching enzymes, strong mechanical properties of chorions, environmental hypoxia, and weakened spontaneous movement of embryos may generally lead to delayed or failed embryo hatching^16^. Embryo development is delayed, and thus results in disturbed hatching, and/or chemical exposure induced deformations, which could impede movement^17^. Regardless of the reason, a failure in hatching usually leads to a lethal outcome.

Furthermore, our studies have demonstrated that ETZ induced a series of cardiac-related deformities in the larva, suggesting an influence on cardiac development and function. A statistically significant decrease of heartbeat and pericardial edema at concentrations of 25 µM and 50 µM, respectively observed at 96 hpf was noted. Additionally, AO staining revealed death cells of myocardial tissue after ETZ 50 µM exposure. As the highest dose (200 µM) caused 87% decrease in a heartbeat compared to the control, we may suggest that the recorded bradycardia indicates a strong cardiotoxic potential of ETZ. As the heart is the first functional organ that is formed during *D. rerio* development, ETZ-cardiotoxicity can be attributed to disruptions in one or more developmental stages. However, we may also consider it as a part of general toxicity, as it is well known that one of the side effects of opioids is induction of the cardiovascular ailments. In both, opioid-consuming patients and abusers, prolongation of the QT interval, which can lead to torsades de pointes, is the potential of causing sudden death^18^. Although *D. rerio* presents a prototypic vertebrate heart with a single atrium and ventricle, the mechanisms of its activity appear to be similar to higher vertebrates, especially that more than 95% of drugs-inducing QT prolongation in humans have the same outcomes in ZF model^5^. In the present study after 40 min. of acute incubation with ETZ, we noticed bradycardia of both chambers with regularly beating heart (1:1) at lower concentrations (50 and 100 µM), while bradycardia and 2:1 atrioventricular block occurred after exposure on the highest concentrations (200 and 300 µM). Extended exposure time or higher concentration can lead to a ventricular beat arrest or cardiac arrest. So far, many substances mainly zERG (*D. rerio* ether-a-go-go-related gene) channel inhibitors and calcium channel activators have been identified as inducing ventricular arrhythmias in the 2:1 and higher degree from in the ZF model^19,20^. As regards to ETZ, the cardiotoxicity mechanisms require further investigation, but we may speculate that these abnormalities may develop also in humans. Therefore, the obtained data should be taken into account by physicians treating ETZ intoxication.

By comparing toxicological outcomes (Fig. S2), we noticed that MORPH is ~ 135 times less toxic than ETZ in *D. rerio* FET assay. Determined LC_50_ = 25.21 mM for MORPH stays in line with previous studies (LC_50_ = 23.39 mM) carried out in FET under static exposure^21^. Contrary to the findings of Sanchez^12^, we did not note any malformations, nor consequences on the heart in nanomolar range treated larvae. However, it is difficult to discuss obtained results in the context of the abovementioned studies as the information on used MORPH salt and purity was not provided. In our studies MORPH-treated larvae in higher concentration—20 mM exhibit heart edema and show slightly less impact a heartbeat (14%, highest concentration vs negative control) in comparison to the strong cardiotoxic effect of ETZ. No influence on the hatching process was observed after incubation in MORPH solution. The evaluation of toxicological endpoints at 96 hpf of ETZ vs MORPH (Fig. S2) exposed a different pattern of action. It should be noted that the appearance of cardiac edema is preceded by a significant hatching inhibition in ETZ incubated larvae. At the highest concentration of the drug, mortality is a consequence of emerging cardiac dysfunctions in combination with general malformations of the body and inhibition of development. The toxicological profile of MORPH observed in our studies significantly differs from ETZ. The hatching process does not affect developmental toxicity, while the appearance of malformations coincides with an increase in mortality. Based on the chemical structure and similarity to known opioids, we suggest that observed ETZ-induced developmental abnormalities are related to activation of opioid system. During embryogenesis, the *D. rerio* opioid system plays an important role, particularly in neurodevelopment, proliferation, and differentiation^22^. Studies suggest that different opioids receptors are involved in a specific process during development. It was revealed that the opioid receptor (zfMOR) mRNA was detected at higher levels than the other opioid receptors during the segmentation period (up to 24 hpf), when the CNS is beginning its subdivision and differentiation into the complex structure^23^. Microarray analyses after chronic exposure to 10 nM MORPH from 5 to 24 hpf identified alteration of several genes associated with µ opioid receptor expression and genes involved in neuronal development, CNS patterning processes, neuronal differentiation, dopaminergic neurotransmission, the serotoninergic signaling pathway, and glutamatergic neurotransmission^24^.

Since the structure of the studied substance can affect its physicochemical properties, lipophilicity, and pharmacokinetic, it is also crucial to take its impact into consideration. Thus, to better understand the observed toxicological differences between tested substances, we performed an ADME in silico analysis. As compound uptake and its toxicity are likely to be influenced by lipophilicity (logP), chemicals with higher logP, and thus more lipophilic, tended to be more often toxic to the developing embryos than chemicals with lower logP^25^. Accumulation of highly lipophilic compounds in *D. rerio* embryos has been previously described^26^. The predicted clogP of ETZ is significantly higher than MORPH and thus makes the drug able to easily penetrate biological membranes including chorion, and accumulate in the larva’s body. The results imply that the greater toxicity of ETZ towards MORPH is associated with its lipophilic character, which is in line with the aforementioned literature. Following further, in a toxicological context, the effect on Pgp should be also considered. This protein is a member of the ATP-binding transmembrane glycoprotein family [ATP-binding cassette (ABC)], which can excrete drugs from inside the cells to outside the surrounding^27^. In accordance with our in silico profiling, according to ADME prediction, ETZ does not meet the criteria of the P-gp substrate, whereas MORPH has been already identified as P-gp substrate in cell culture system^28^, through radiolabeling and in P-gp KO mice^29^.

Taken together with other predicted properties, that is high GIA, and the possibility to cross BBB, ETZ is a potential neurotoxicant. This assumption is supported by the fact that analysis of post-mortem samples revealed a high accumulation of isotonitazene in the brain and pericardial fluids^30^. This compound is another benzimidazole-derived opioid analgesic drug related to ETZ, and was characterized as stronger than ETZ (MOR-βarr2, EC_50_ = 1.63 nM; MOR-mini-Gi, EC_50_ = 3.72 nM). Interestingly, its metabolite N-desethylisotonitazene presents higher potency (MOR-βarr2, EC_50_ = 0.614 nM; MOR-mini-Gi, EC_50_ = 1.16 nM)^13^ , which may intensify or be the major reason the toxicity effect. In the context of present studies identification of ETZ metabolites seems to be crucial. Therefore, since the *D. rerio* model is successfully used to identify metabolites, our investigation based on this model revealed the creation of one metabolite by N-dealkylation process. For isotonitazene, four metabolites in human blood and urine specimens were found, and authors suggest that the N-dealkylation and/or O-dealkylation biotransformation may occur in the other benzimidazole-based opioid analogs^31^. Thus, our results lead to a similar conclusion N-desalkyl species are appropriate metabolite biomarkers for routine screening of biological specimens. Thus, confirmation of the metabolite presence and its structure elucidation is important for further research as the findings presented by us, and discoveries of strong pharmacological potential of 2-benzylbenzimidazole family metabolites by Vandeputte^13^, highlight a possible major contribution of metabolite or synergism with the parent compound in toxicological, and especially cardiotoxic outcomes.

Another crucial factor for assessing metabolic toxicity is the interaction with cytochrome P450s enzymes, which are produced in the liver and are involved in phase I of metabolism. In silico profiling revealed that ETZ is a possible inhibitor of 1A2, 2C19, 2D6, and 3A4 subfamilies. Inhibition of CYPs impairs the biotransformation or clearance of many substances resulting in different half-life, higher plasma levels of drugs, and possible drug-drug interactions within multidrug users. Many NPS were already identified as substrates of mentioned isozymes. Methylone is metabolized primarily by CYP2D6 with less contribution of CYP1A2 and 2C19^32^, mephedrone by CYP2D6^33^, 4F-MDMB-BINACA by CYP: 1A2, 2C19, 3A4^11^. Recently CYP3A4 and CYP2D6 were found as enzymes involved in two fentanyl derivates, 4F-Cy-BAP and Fu-BAP, pathways of metabolism^9^.

In conclusion, our studies showed severe dose-dependent developmental toxicity of ETZ—cardiotoxicity, increase in mortality, developmental malformations and a delayed hatching process are more pronounced than in well-known opioid—MORPH. Further, phase I metabolite of ETZ resulting from N-dealkylation reaction was identified, and therefore it should be considered as a target for toxicological screening. In silico findings indicate the high toxic potential of ETZ, as it has been identified as an inhibitor of several CYPs (1A2, 2C19, 2D6, 3A4) which may lead to drug-drug interactions and accumulation of substances. Nonetheless, the exact mechanism of observed effects in response to ETZ should be examined further. In the next step evaluation of metabolites’ properties of as well as their interactions with parent compound are necessary.

## Materials and methods

### Chemicals and reagents

MORPH hydrochloride (7,8-Didehydro-4,5-epoxy-17-methyl-(5α,6α)-morphinan-3,6-diol hydrochloride; PubChem CID: 5,464,110) was obtained from Polfa Kutno, Poland. ETZ (2-[2-[(4-ethoxyphenyl)methyl]benzimidazol-1-yl]-*N*,*N*-diethylethanamine; PubChem CID: 149,797,386) was provided for research purposes by the Institute of Toxicology Research (Borowa, Poland) as a part of actions by the Polish police. Identification and purity check was done by X-ray crystallography, ^1^H and ^13^C spectroscopies, LC–MS/MS and GC–MS chromatography, UV–VIS and FT-IR spectroscopy techniques and already has been published in previous work^34^. Acetonitrile, methanol, H_2_O formic acid, all LC–MS grades were purchased from Sigma-Aldrich (Germany).

### *D. rerio* culture and embryo toxicity test (FET)

*D. rerio* of the AB strain (Experimental Medicine Centre, Medical University of Lublin, Poland) were maintained at 28.5 °C, on a 14/10 h light/dark cycle, under standard aquaculture conditions (pH of water 6.9–7.5, conductivity 500–800 µS, average daily water change cycle 10–15%). Fertilized eggs were collected via natural spawning. Embryos were reared in E3 embryo medium (pH 7.1–7.3; 17.4 µM NaCl, 0.21 µM KCl, 0.12 µM MgSO_4_ and 0.18 µM Ca(NO_3_)_2_) in an incubator (IN 110 Memmert GmbH, Germany) at 28.5 °C. The FET test was performed based on OECD Guidelines for the Testing Chemicals, Test No. 236 with few modifications. Stock and dilutions of MORPH and ETZ were prepared in E3 embryo medium freshly. On average, thirty embryos (0 hpf) were incubated at each concentration (10, 25, 50, 75, 100, 125, 150, 200, 300 µM) of ETZ or MORPH (1, 10, 20, 25, 30, 50 mM) in 6 well-plates, based on range-finding study. Intact embryos were chosen, transferred individually to 96-well plates, then the exposure was continued with fresh solution for a period of 96 h. The experiment was conducted in a static environment (no solution changing, as recommended in Test no 236 of OECD: measured ETZ and MORPH concentrations before and after incubation were within the 20% range.Dead larvae were removed from the plate, while the remaining larvae stayed in their wells until the end of the experiment, then larvae were sacrificed by 15% tricaine overdose. After 48, 72, 96 hpf toxicological endpoints were checked: lack of somites, hatchability, abnormal body formation, coagulation. Heartbeats per minute were counted after 96 hpf exposure at 10 randomly selected larvae per concentration after 30 min of habituation to microscope light and room temperature. Pictures (magnification 3.2x) were captured using stereoscopic microscope Stereo Discovery V8 (Carl Zeiss Microscopy GmbH, Germany). All drug exposure tests were done with 24 larvae per concentration in three independent replications, making a total of 72 in each group (total in whole FET n = 1152).

All experiments were conducted following the National Institute of Health Guidelines for the Care and Use of Laboratory Animals and to the European Community Council Directive for the Care and Use of Laboratory Animals of 22 September 2010 (2010/63/EU)—applies in Poland. For the experiment with larvae up to 5-dpf, agreement of the Local Ethical Commission is not required. After each experiment, larvae were immediately anesthetized and sacrificed with 15% tricaine. The experiment design was adhered to the ARRIVE guidelines for reporting animal research^35^.

### Quantification of ETZ and MORPH in post-exposed solution

Sample was diluted with methanol and 10 μL was transferred to a 150 μL vial with a cap containing 80 μL of methanol and 10 μL of internal standard (IS) (4-MMC-*d*_*3*_, 1 μg/mL). Solution was transferred to silanized glass insert and analyzed by UPLC-QQQ-MS with already developed method^34^.

### In vivo cell death assay

The mechanism of cellular toxicity was determined in live *D. rerio* using acridine orange (AO) staining, which is a nucleic acid selective metachromatic dye. Three randomly selected embryos (4 dpf) after FET experiment were incubated with 5 µg/ml of AO for 1 h at 28 °C. Then, embryos were washed in E3 buffer and mounted in methylcellulose. Apoptotic cells were captured using a fluorescence microscope and the fluorescence intensity of apoptotic cells in the heart region was quantified using ImageJ.

### Cardiac rhythm analysis

Five, new *D. rerio* embryos at 4 dpf stage of development (total n = 75; from three independent experiments) were pooled at 6 well plate per well in E3 solution and habituated for 30 min to miscroscope light and room temperature. Then solution was changed to 1 ml of ETZ at different concentrations (10, 50, 100, 200, 300 µM) and incubated up to 40 min. Concentrations and time were determined through previous experiments. The plate with the embryos was kept on the microscope during the incubation time. Every 10 min. heart region of each embryo was recorded at 30 fps, 1080p for 12 s. by a camera installed onto microscope ocular. Videos processing were done according to the already published procedure^36^. VirtualDub 1.10.4 was used to extract 10 s (300 frames) and convert it into uncompressed AVI format. Obtained files were opened by ImageJ 1.53, then the atrium and ventricle region was selected, subsequently using “plot z-axis profile” with “live” option, the time profile of pixel intensity change (as beat) was generated. These values were plotted using OriginPro 9.0, adjacent averaging (points of window 5, without weighted average) smoothing method with automatic positive peak detection was applied. The total number of positive peaks is equal to the number of atrium or ventricle beats per 10 s. Beat-to-beat interval was calculated as the difference between the next and previous peak values.

### In silico ADME profiling

The ADME-related physicochemical properties of ETZ and MORPH were predicted by the SwissADME online Web server^37^. Consensus logP (logarithm of compound partition coefficient between n-octanol and water), as an average of different model predictions (iLOGP, XLOGP3, WLOGP, MLOGP, SILICOS-IT), was calculated. In addition, the BOILED-Egg model of tested compounds was predicted to show the capability of gastrointestinal (GI) absorption and permeability of the blood–brain barrier (BBB). Bioavailability score was evaluated according to certain rules, Lipinski’s rule of five, Ghose, Veber, Egan, Muegge. Furthermore, possible interactions with major human cytochrome P450 (CYP) isoforms involved in drug metabolism, 1A2, 2C19, 2C9, 2D6, 3A4 were generated.

### Ex vivo identification of metabolites

In compliance with an already described procedure^8^ for metabolism studies, ten *D. rerio* larvae at 4 dpf (total n = 40) were pooled in a 6-well plate containing 3 ml of 75 μM ETZ. Concentration was chosen based on maximum tolerated concentration (MTC) results performed according to Richter et al. 2019^8^. After 24 h of exposure at 28 °C, larvae, and medium were collected. Three independent repetitions were performed (total n = 30). After the experiment, larvae were immediately anesthetized with 15% tricaine and sacrificed by decapitation. To confirm the absence of interfering compounds, a blank group of *D. rerio* larvae without substance were prepared. Furthermore, a control medium sample containing only the drug was prepared, for the detection of compound degradations during the incubation step. For precipitation to 50 µl of homogenate, 200 µl of cold ACN with 0.1% (v/v) HCOOH was slowly added and vortexed. Subsequently, samples were sonicated for 15 min., at + 4 °C. After that tubes with solution were mounted in a rack, and the solvent was evaporated at room temperature using nitrogen stream. The content was dissolved in 30 µl of MeOH and transferred to a vial with insert mounted. 5 µl was injected onto the HPLC-HRMS/MS system as described below.

### LC–MS/MS system

Analysis was performed using HPLC/ESI-QTOF-MS system in positive ion mode with a 630B accurate mass QTOF-MS (Agilent Technologies INc., Santa Clara, CA, USA) mass spectrometer and an ESI-Jet-Stream ion source. The separation was done using Gemini 100 × 2.1, 3.5 µm column, thermostated at + 40 °C. The mobile phase consisted of a mixture of H_2_O + 0.1% HCOOH (A) and ACN + 0.1% HCOOH (B). The gradient elution was carried out at a constant flow of 0.4 mL/min. The gradient applied was as follows 0 min., 5%B; 15 min., 98%B; 20 min., 98%B. The return to the initial gradient compositions (95% A/5% B) was held for 5 min. The total time of analysis was 20 min. ESI-QTOF-MS analysis was performed with the following parameters, gas (N_2_) temperature: 300 °C, flow rate 10 mL/min, 35 psig, sheath gas temperature: 325 °C, flow rate 10 L/min, fragmentor voltage 140 V, Vcap 4000 V. Two collision energies, 20 and 40 eV were chosen. Data acquisition was performed in Auto MS/MS mode at the range of 100–1000 mass units. Mass Hunter B.08.00 software (Agilent Technologies INc., Santa Clara, CA, USA) was used for data analysis. Mass calibration was done before analysis according to the manufacturer’s recommendations to ensure mass accuracies below 5 ppm. Data handling was performed by MassHunter Workstation Software B.08.00 and OriginPro 9.5.

### Statistical analyses

The median lethal concentration (LC_50_) was determined by nonlinear, four parameters regression analysis. Obtained data were tested with the use of Shapiro–Wilk and Brown-Forsythe test. The results were processed by the one-way ANOVA analysis with Dunnett’s post-test or two-way ANOVA analysis with Tukey’s test. A *p*-value < 0.05 was considered statistically significant. All statistical analyses were performed using GraphPad Prism 8.

### Ethical approval

All experiments were conducted in accordance with the National Institute of Health Guidelines for the Care and Use of Laboratory Animals and to the European Community Council Directive for the Care and Use of Laboratory Animals of 22 September 2010 (2010/63/EU). For the experiment with larvae up to 5-dpf, the agreement of Local Ethical Commission is not required.

## Supplementary Information

Supplementary Information 1.

Supplementary Video S1.

Supplementary Video S2.

## Data Availability

The data presented in this study are available on request from the corresponding author.

## References

[1] EMCDDA. European Drug Report: Trends and Developments. (2020). 10.2810/123451.

[2] Lamy FR (2021). “Etazene, safer than heroin and fentanyl”: Non-fentanyl novel synthetic opioid listings on one darknet market. Drug Alcohol Depend..

[3] Morais, J. De *et al*. EMCDDA technical report on the new psychoactive substance N,Ndiethyl- 2-[[4-(1-methylethoxy)phenyl]methyl]-5-nitro-1Hbenzimidazole- 1-ethanamine (isotonitazene). 1–40 (2020).

[4] Hunger A, Kebrle J, Rossi A, Hoffmann K (1960). Benzimidazol-derivate und verwandte heterocyclen. II. Synthese von 1-Aminoalkyl-2-benzyl-benzimidazolen. Helv. Chim. Acta.

[5] Cassar S (2020). Use of zebrafish in drug discovery toxicology. Chem. Res. Toxicol..

[6] Howe K (2013). The zebrafish reference genome sequence and its relationship to the human genome. Nature.

[7] Ninkovic J, Bally-Cuif L (2006). The zebrafish as a model system for assessing the reinforcing properties of drugs of abuse. Methods.

[8] Richter LHJ (2019). Tools for studying the metabolism of new psychoactive substances for toxicological screening purposes: A comparative study using pooled human liver S9, HepaRG cells, and zebrafish larvae. Toxicol. Lett..

[9] Gampfer TM (2020). Toxicokinetics and toxicodynamics of the fentanyl homologs cyclopropanoyl-1-benzyl-4´-fluoro-4-anilinopiperidine and furanoyl-1-benzyl-4-anilinopiperidine. Arch. Toxicol..

[10] Prado E (2021). Metabolism of synthetic cathinones through the zebrafish water tank model: A promising tool for forensic toxicology laboratories. Forensic Toxicol..

[11] Wagmann L (2020). How to study the metabolism of new psychoactive substances for the purpose of toxicological screenings: A follow-up study comparing pooled human liver S9, HepaRG cells, and zebrafish larvae. Front. Chem..

[12] Sanchez-Simon FM, Arenzana FJ, Rodriguez RE (2010). In vivo effects of morphine on neuronal fate and opioid receptor expression in zebrafish embryos. Eur. J. Neurosci..

[13] Vandeputte MM (2021). Synthesis, chemical characterization, and μ-opioid receptor activity assessment of the emerging group of ‘nitazene’ 2-benzylbenzimidazole synthetic opioids. ACS Chem. Neurosci..

[14] Luethi D, Liechti ME (2020). Designer drugs: Mechanism of action and adverse effects. Arch. Toxicol..

[15] Fuiman, L. A. & Werner, R. G. Chapter 1: Special Considerations of Fish Eggs and Larvae. In Fishery science. The Unique Contributions of Early Life Stages (Blackwell Science, 2002).

[16] Jezierska B, Ługowska K, Witeska M (2009). The effects of heavy metals on embryonic development of fish (a review). Fish Physiol. Biochem..

[17] Ong KJ (2014). Mechanistic insights into the effect of nanoparticles on zebrafish hatch. Nanotoxicology.

[18] Behzadi M, Joukar S, Beik A (2018). Opioids and cardiac arrhythmia: A literature review. Med. Princ. Pract..

[19] Letamendia A (2012). Development and validation of an automated high-throughput system for zebrafish in vivo screenings. PLoS One.

[20] Langheinrich U, Vacun G, Wagner T (2003). Zebrafish embryos express an orthologue of HERG and are sensitive toward a range of QT-prolonging drugs inducing severe arrhythmia. Toxicol. Appl. Pharmacol..

[21] Ali S, van Mil HGJ, Richardson MK (2011). Large-Scale assessment of the zebrafish embryo as a possible predictive model in toxicity testing. PLoS One.

[22] Bao W (2019). Opioid neurobiology, neurogenetics and neuropharmacology in zebrafish. Neuroscience.

[23] Macho Sanchez-Simon F, Rodriguez R (2008). Developmental expression and distribution of opioid receptors in zebrafish. Neuroscience.

[24] Herrero-Turrión MJ, Rodríguez-Martin I, López-Bellido R, Rodríguez RE (2014). Whole-genome expression profile in zebrafish embryos after chronic exposure to morphine: Identification of new genes associated with neuronal function and mu opioid receptor expression. BMC Genomics.

[25] Padilla S (2012). Zebrafish developmental screening of the ToxCast^TM^ Phase I chemical library. Reprod. Toxicol..

[26] de Koning C (2015). Visualizing compound distribution during zebrafish embryo development: The effects of lipophilicity and DMSO. Birth Defects Res. Part B—Dev. Reprod. Toxicol..

[27] Schinkel AH (1999). P-Glycoprotein, a gatekeeper in the blood-brain barrier. Adv. Drug Deliv. Rev..

[28] Callaghan R, Riordan JR (1993). Synthetic and natural opiates interact with P-glycoprotein in multidrug- resistant cells. J. Biol. Chem..

[29] Schinkel AH, Wagenaar E, Van Deemter L, Mol CAAM, Borst P (1995). Absence of the mdr1a P-glycoprotein in mice affects tissue distribution and pharmacokinetics of dexamethasone, digoxin, and cyclosporin A. J. Clin. Invest..

[30] Mueller F (2021). Isotonitazene: Fatal intoxication in three cases involving this unreported novel psychoactive substance in Switzerland. Forensic Sci. Int..

[31] Krotulski AJ, Papsun DM, Kacinko SL, Logan BK (2020). Isotonitazene quantitation and metabolite discovery in authentic forensic casework. J. Anal. Toxicol..

[32] Pedersen AJ, Petersen TH, Linnet K (2013). In vitro metabolism and pharmacokinetic studies on methylone. Drug Metab. Dispos..

[33] Pedersen AJ, Reitzel LA, Johansen SS, Linnet K (2013). In vitro metabolism studies on mephedrone and analysis of forensic cases. Drug Test. Anal..

[34] Siczek M, Zawadzki M, Siczek M, Chłopaś-Konowałek A, Szpot P (2020). Etazene (N, N-diethyl-2-{[(4-ethoxyphenyl)methyl]-1H-benzimidazol-1-yl}-ethan-1-amine (dihydrochloride)): A novel benzimidazole opioid NPS identified in seized material: crystal structure and spectroscopic characterization. Forensic Toxicol..

[35] du Sert NP (2020). The ARRIVE guidelines 2.0: Updated guidelines for reporting animal research. PLOS Biol..

[36] Gaur H (2018). ZebraPace: An open-source method for cardiac-rhythm estimation in untethered zebrafish larvae. Zebrafish.

[37] SwissADME. SwissADME. Available at: http://www.swissadme.ch/. (Accessed: 15th March 2021)

