# Supplementary material for: Etazene induces developmental toxicity in vivo Danio rerio and in silico studies of new synthetic opioid derivative
Source: Sci Rep. 2021 Dec 20;11:24269. doi: 10.1038/s41598-021-03804-9 (PMC8688443; doi:10.1038/s41598-021-03804-9)
Supplement: Supplementary file 1 — Supplementary Information 1. [file 41598_2021_3804_MOESM1_ESM.docx]

**Supplementary Materials**

**Etazene induces developmental toxicity *in vivo Danio rerio* and *in silico* studies of new synthetic opioid derivative**

Łukasz Kurach^1*^, Agnieszka Chłopaś-Konowałek^2^, Barbara Budzyńska^1^, Marcin Zawadzki^3^, Paweł Szpot^3^, Anna Boguszewska-Czubara^4^

^1^ Independent Laboratory of Behavioral Studies, Medical University of Lublin, 4A Chodzki Str., 20-093 Lublin, Poland

^2^ Institute of Toxicology Research, 45 Kasztanowa Str.,55-093 Borowa, Poland

^3^ Department of Forensic Medicine, Wroclaw Medical University, 4 J.Mikulicza-Radeckiego Str., 50-345 Wrocław, Poland

^4^ Department of Medical Chemistry, Medical University of Lublin, 4A Chodzki Str., 20-093 Lublin, Poland

*Correspondence: Łukasz Kurach, e-mail: lukasz.kurach@umlub.pl, tel. +48 814486196


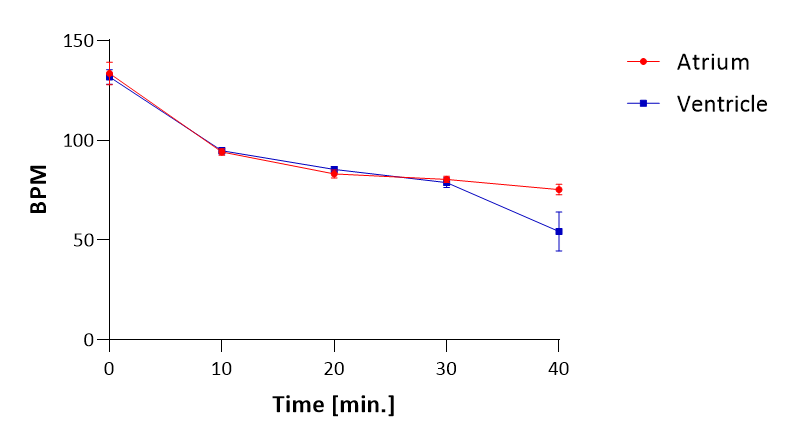


Figure S1. Evaluation of cardiac rhytm after 200 µM ETZ exposition 0 – 40 min; n = 3 from three independent experiments.


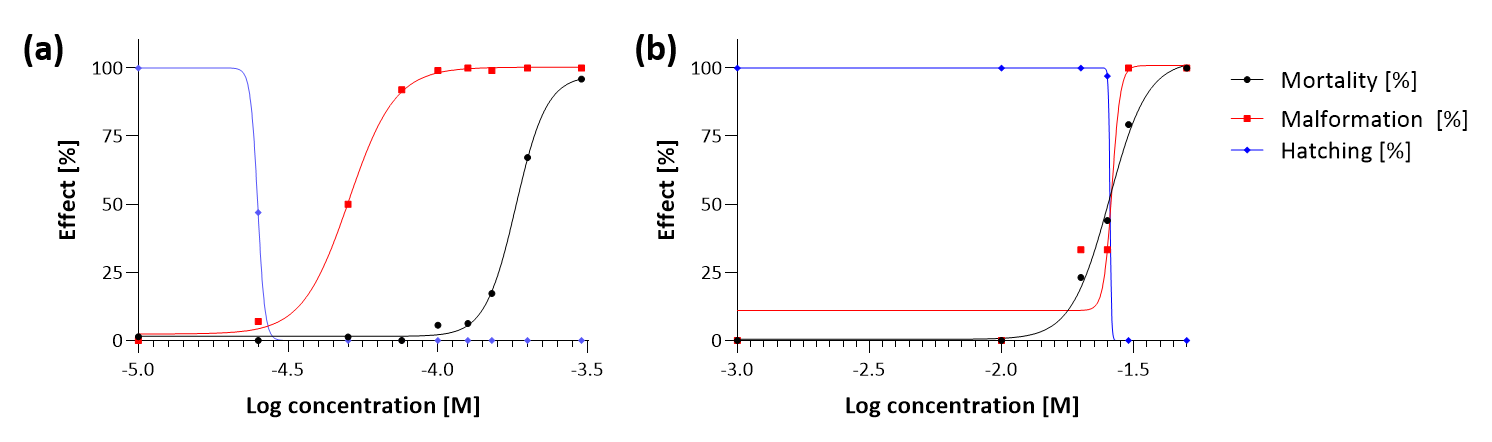


Figure S2. Relationship between observed morphological changes namely malformation rate, hatching and mortality rate at last day of exposure (96 hpf) for (a) ETZ, (b) MORPH.

Video captions

Video S1. Representative heartbeat video of negative control.

Video S1. Representative heartbeat video after 40 minutes and 200 µM ETZ treatment.
